# *Streptococcus agalactiae* is not always an obligate intramammary pathogen: Molecular epidemiology of GBS from milk, feces and environment in Colombian dairy herds

**DOI:** 10.1371/journal.pone.0208990

**Published:** 2018-12-10

**Authors:** Claudia Cobo-Ángel, Ana S. Jaramillo-Jaramillo, Laura M. Lasso-Rojas, Sandra B. Aguilar-Marin, Javier Sanchez, Juan C. Rodriguez-Lecompte, Alejandro Ceballos-Márquez, Ruth N. Zadoks

**Affiliations:** 1 Research Group in Milk Quality and Veterinary Epidemiology, Faculty of Agricultural Sciences, Universidad de Caldas, Manizales, Colombia; 2 Atlantic Veterinary College, University of Prince Edward Island, Charlottetown, Prince Edward Island, Canada; 3 Institute of Biodiversity, Animal Health and Comparative Medicine, College of Medical, Veterinary and Life Sciences, University of Glasgow, Glasgow, United Kingdom; 4 Moredun Research Institute, Penicuik, United Kingdom; University of Illinois, UNITED STATES

## Abstract

For many years *Streptococcus agalactiae* has been considered an obligate intramammary and strictly contagious pathogen in dairy cattle. However, recent reports of *S*. *agalactiae* isolation from extramammary sources have contradicted that premise. To gain further insight into the epidemiology of *S*. *agalactiae* infection in cattle, we examined its distribution and heterogeneity of strains in bovine milk, bovine feces, and the environment in Colombian dairy farms. First, a longitudinal study was conducted at herd level in 152 dairy herds. Bulk tank milk samples from each herd where collected twice a month for six months. A follow-up study with a cross sectional design at the cow level was conducted in a subset of 25 farms positive for *S*. *agalactiae*. Cow-level milk samples from 1712 lactatting cows and 1545 rectal samples were collected, as well as 120 environmental samples. Samples were used for *S*. *agalactiae* detection and genotyping using Multi Locus Sequence Typing. Results showed sporadic rather than repeated isolation of *S*. *agalactiae* from bulk tank milk in 40% of the positive herds, challenging the idea that *S*. *agalactiae* is a highly contagious pathogen causing chronic infections. *S*. *agalactiae* was isolated from rectal or environmental samples in 32% and 12% of cross-sectional study farms, respectively, demonstrating that the bacteria can survive in extramammary sources and that *S*. *agalactiae* is not an obligate intramammary pathogen. The same strain was isolated from rectal and bulk tank milk samples in eight farms, suggesting that fecal shedding is frequent, and contributes to the presence of *S*. *agalactiae* in bulk tank. High within-herd heterogeneity of strains was found, which is distinct from the situation in developed dairy industries. These new epidemiological findings should be considered to adjust surveillance and control recommendations for *S*. *agalactiae*.

## Introduction

*Streptococcus agalactiae* is a Gram-positive coccus, facultatively aerobic and encapsulated, also known as Group B Streptococcus (GBS) based on its Lancefield classification [1]. Group B Streptococcus can infect multiple hosts including humans and bovines, among others [2]. It is an important cause of neonatal infections in humans [3]. In adults, GBS colonization in throat, gastrointestinal and genitourinary tracts is common whilst GBS is also increasingly recognized as an invasive pathogen in adults worldwide [4, 5]

In bovines, GBS is a major mastitis pathogen. It causes intramammary infection, which is usually subclinical and chronic, often contagious and with low probability of self-cure [6]. Bovine mastitis affects milk quality and milk quantity, threatening the dairy industry’s competitiveness. It is one of the most common and costly diseases of dairy cattle, and causes economic losses related to decrease in milk production, costs of veterinary assistance and treatments, milk discards, early culling of animals and elevated somatic cell count (SCC) [7]. Somatic cell count is legally regulated in Europe and North America and used as the basis for premiums or penalties [8]. In countries where there is no penalty for high SCC, such as Colombia, economic losses due to mastitis have not been quantified, but they can be related to decreased milk production, early culling of chronically infected animals, and high bacteria counts, with penalties on the milk price when the total bacteria count is higher than 200.000 colony forming units (CFU)/mL [9].

The prevalence of GBS in dairy herds in countries in Europe and North America, where long-standing mastitis control programs have been stablished, is less than 10% [10, 11]. However, GBS is still a threat for many countries, particularly for those with developing dairy industries such as Colombia and Brazil, where herd prevalence is 40% and 60%, respectively [12, 13]. Other countries with high herd-level GBS prevalence include Spain (36%) [14], Germany (29%) [15], and China (92%) [16]. Some countries that had successfully controlled GBS, have reported the re-emergence of this pathogen, e.g. Denmark and Norway [10, 17]. Group B Streptococcus re-emergence is probably driven by a combination of host, environment (management) and pathogen factors, showing changes in its epidemiology, adaptability or survival skills.

For many years, GBS has been considered an obligate intramammary and strictly contagious pathogen in dairy cattle, and control plans were established successfully based on that premise [8]. However, not all GBS is shed or transmitted at the same level [18, 19]. Moreover, GBS has also been isolated from the gastrointestinal tract of cows and environmental samples, suggesting a potential oral-fecal transmission cycle and explaining the failure of traditional control measures [17, 20]. Finally, the distribution of strains differs between countries, providing further evidence that one size may not fit all in understanding and preventing GBS transmission [17, 21, 22]. To improve current control plans for GBS, it is necessary to understand the within herd molecular epidemiology, existence of extramammary sources and the possible transmission routes. Considering the above, and to gain further insight into the epidemiology of GBS disease in cattle, we examined the distribution and heterogeneity of GBS strains in bovine milk, bovine feces, and the farm environment in Colombian dairy herds.

## Materials and methods

### Ethics statement

This study was approved by the Bioethics Committee for Animal Experimentation of Universidad de Caldas (Document: 130705B-15). Owners of the farms selected for conducting this work gave their permission to carry out the study. No additional permissions for sample collection and analysis were needed.

### Study design

First, a longitudinal study was conducted at herd level on 152 commercial dairy farms with unknown GBS history to identify GBS positive herds and to explore its dynamics in bulk tank milk (BTM). Then, a second study was conducted with a cross sectional design at the cow level in a subset of 25 GBS positive farms selected from the longitudinal study. The characteristics of the herds included in both studies, herd management and milking practices are summarized in Table 1.

**Table 1:** Herd management and milking practices of 152 herds participating in a longitudinal study of *Streptococcus agalactiae* in bulk tank milk, and 25 herds participating in a cross-sectional study in central western Colombia.

Herd characteristics	Longitudinal study (n = 152)	Cross sectional study (n = 25)
Median herd size (including young stock and dry cows)	80 (range: 3–398)	77 (range: 19–336)
Median lactating cows	44 (range: 3–210)	74 (range 11–156)
Median milk production (kg/cow/d)	14.2 (range: 5–25)	14.5 (Range: 8–25)
*Production system*		
Rotational grazing only	15 (10%)	1 (4%)
Rotational grazing + supplementation	135 (89%)	24 (96%)
Extensive (i.e. No rotational grazing and no supplementation)	2 (1%)	0 (0%)
*Biosecurity*		
Closed herd	99 (65%)	7 (28%)
Use of California Mastitis Test for mastitis diagnosis	143 (94%)	25 (100%)
Defined policies about culling for mastitis	22 (14%)	5 (20%)
Segregate cows with mastitis	27 (18%)	0 (0%)
*Milking practices*		
Use of gloves during milking	33 (22%)	8 (32%)
Pre-milking teat disinfection	113 (74%)	22 (88%)
Post milking teat disinfection	136 (89%)	23 (92%)
Dry cow therapy	147 (97%)	24 (96%)
*Milking frequency*		
1x/day	8 (5%)	0 (0%)
>1x/day	144 (95%)	25 (100%)
*Milking method*		
Hand	71 (47%)	8 (32%)
Mechanical	79 (52%)	15 (60%)
Mixed	2 (1%)	2 (8%)
*Milking location*		
Parlor	63 (41%)	11 (44%)
Pen	43 (28%)	6 (24%)
Paddock	46 (30%)	8 (32%)

### Longitudinal study

Between January 2013 and November 2014, 152 commercial dairy herds were selected in three departments from the central western region of Colombia: 77 herds in Caldas, 46 herds in Risaralda and 29 herds in Quindío.

Two bulk tank milk (BTM) samples from each herd were collected twice a month for six months. Samples were collected using a metallic scoop, which was disinfected with 70% ethanol and flamed prior to use. Samples were collected in sterile 30 mL vials, one with bronopol as preservative for bulk tank SCC (BTSCC) analysis, and one with no preservatives for microbiological culture. Samples were labeled with a unique herd code assigned by the milk processor, location and date. An internal laboratory code was assigned for those herds not delivering milk to a processing plant. Afterwards, BTM samples were refrigerated and transported to the milk quality laboratory of Universidad de Caldas. Information about herd management and lab testing was recorded in Epidata V 2.0 (The EpiData Association, Odense, Denmark).

Bulk tank SCC was determined using a MilkoScan FT2 milk analyzer (Foss Electric, Hillerød, Denmark). For microbiological culture, an aliquot of 0.01 mL of each milk sample was inoculated on Edward’s agar media and incubated at 37° for 24–48 hours. Esculin-negative colonies were selected for Christie, Atkins, Munch-Petersen (CAMP) reaction as follows: each colony was cultured on blood-esculine agar with 5% washed bovine blood, crossed with a streak of *Staphylococcus aureus* (ATCC 25923), and aerobically incubated at 37°C for 24 hours. All plates were streaked with a positive (*S*. *agalactiae* ATCC 27956) and negative control (*S*. *uberis* ATCC 700407). To avoid false-positive samples, CAMP positive colonies were confirmed as *S*. *agalactiae* by a GBS-specific PCR reaction using primers STRA-AgI (AAGGAAACCTGCCATTTG) and STRA-AgII (TTAACCTAGTTTCTTTAAAACTAGAA), which targets the 16S-23S intergenic spacer region, with a product of 270 bp [23].

Culture results and management recommendations were shared with individual herd owners at the end of the six-month sampling period.

### Cross sectional study

Composite milk samples were collected from all lactating cows (n = 1712) in a subset of 25 herds in Caldas between July 2016 and May 2017. Herds were selected based on their GBS positivity in the BTM samples from the longitudinal study. After udder preparation for milking, teat ends were disinfected with 70% ethanol and the first milk strips were discarded. An approximately equal volume of milk from each quarter was collected into a 30 mL sterile vial. For GBS identification, samples were cultured on chromogenic agar media (Strepto B Chrome ID, Biomerieux, Marcy l'Etoile. France) and incubated at 37°C for 24–48 h. One of each morphological type suspicious colony (i.e. colonies from light pink to dark red) was sub-streaked onto blood-esculin agar media to purify it and incubated at 37°C for 24–36 h. Pure colonies were harvested from the blood agar media and suspended in 20 μL of distilled water. Half of this volume was heated in a microwave oven for two minutes and used as a template for species confirmation by colony PCR, following the methodology described for BTM isolates. The remaining ten microliters of diluted colony were inoculated into Todd Hewitt Broth (THB) (BBL, Franklin Lakes, NJ, USA), and incubated at 37°C for 24 h. Isolates were stored at -80°C in Todd Hewitt Broth and glycerol solution at a ratio of 80:20 (v/v), for subsequent molecular characterization.

Additionally, rectal swabs were collected from 1545 cows enrolled for milk sampling. One producer did not give permission for collection of rectal swabs. Samples from the rectum were collected using sterile cotton swabs on wooden sticks, and immediately placed in 2 mL of THB with 0.01 mg/mL colistin sulphate, and 5 μg/mL oxolinic acid (COBA Streptococcus selective supplement, Oxoid, UK). After incubation at 37°C for 24h, enrichments were plated onto the chromogenic media and incubated at 37°C for 24 to 48 h. Species identity of suspect colonies was confirmed by PCR and isolates were stored as described above.

### Environmental sampling

At the end of the milking, samples were collected from the walls and bottoms of the feeders in the milking parlors. Wet areas in feeders where cows had been eating/licking were rubbed with cotton wool swabs, moistened with THB with streptococcus selective supplement COBA (Oxoid, UK). From the 25 herds, 84 swabs were collected with a range of 1 to 8 swabs per herd. Additionally, swabs from the internal walls of 40 drinking water containers were collected in 20 herds (Range: 1–4 per herd) using sterile cotton swabs. All swabs (n = 124) were processed as described for bovine rectal swabs.

### Molecular characterization

Multi-locus sequence typing (MLST) was performed by Streeklab Haarlem (Haarlem, the Netherlands) using the loci described by Jones et al. (2003) and either High-throughput MLST (HiMLST), [24] or next generation sequencing on an Illumina MiniSeq (Illumina, Inc. California, US). For herds that were positive once in the longitudinal sampling, a single isolate was characterized. For herds that were positive more than once, two to four isolates were selected for molecular characterization, including the first and last isolate. For the cross-sectional study, a single isolate per sample was characterized for all GBS positive samples from bovine milk, bovine feces and the farm environment. New allelic profiles were submitted to the database curator for allocation of new allele numbers and STs.

### Data analysis

Descriptive statistics and frequency tables were generated to explore the distribution of data. Differences between frequencies in categorical variables were explored using Pearson *χ*^2^ or Fisher exact testing, as appropriate. The association between GBS detection and BTSCC was explored through a linear mixed model. All variables described in Table 1 were explored as possible predictors, month and quarter of the year when samples were collected and rainfall (mm/month) recorded at the nearest airport weather station were also explored in the models. A repeated measures model of log transformed BTSCC was used (LnBTSCC in thousands/mL; 12 samples per BTM). Correlation structures for repeated measures were explored and a fourth order autoregressive model was used, based on best model fit [25]. Unconditional and multivariable analysis were conducted using this model structure. Variables with P ≤ 0.15 were selected as candidates for the multivariate model. Backward stepwise selection of the variables was made with P ≤ 0.05 as cut-off to be retained in the final model. The variables ‘Province’ and ‘Number of lactating cows’ were explored as potential confounders, but only province resulted as confounder. First-order interactions between all significant variables were explored and significant interactions (P ≤ 0.05) were retained in the final model. Standardized residuals and fitted values were predicted to assess model fit. All statistical analyses were conducted using the software Stata 15 (StataCorp, College Station, Texas, USA).

For molecular characterization data, the complete up-to-date MLST database available on May 5, 2018 (https://pubmLst.org/sagalactiae) was used for a comparative electronic analysis based upon related sequence types, eBURST (http://eburst.mLst.net/) [26], and to create a population snapshot. Closely related STs were assigned to clusters or clonal complexes (CC), using sharing of 5 of 7 alleles to define CCs [27].

## Results

### Longitudinal study

*Streptococcus agalactiae* was found in 327 out of 1809 BTM samples (18.1%), distributed in 72 out of 152 herds (47%). Herd level prevalence was significantly different between provinces (P < 0.001), 62% in Caldas, 35% in Quindío, and 28% in Risaralda. The frequency of GBS isolations ranged from 1 to 12 times per herd. Sporadic isolation (i.e. GBS isolated only once during the study) was found in 29 (40%) out of the 72 positive herds. Fifteen (21%) of the positive herds were GBS positive 2 to 5 times during the study period, 17 (24%) herds were positive 6 to 9 times, and eleven (15%) herds were positive 10 to 12 times. The frequency and proportion of GBS isolation by province is presented in Table 2.

**Table 2:** Frequency and proportion n (%) of *Streptococcus agalactiae* (Group B Streptococcus, GBS) isolation by provinces.

Frequency of GBS isolation	Caldas	Risaralda	Quindio	Total
	n (%)	n (%)	n (%)	n (%)
GBS negative herds	28 (37)	32 (68)	20 (69)	80 (53)
Sporadic (once)	16 (21)	7 (15)	6 (21)	29 (19)
2 to5 times	9 (12)	5 (11)	1 (3)	15 (10)
6 to9 times	12 (16)	3 (6)	2 (7)	17 (17)
10 to12 times	11 (14)	0 (0)	0 (0)	11 (7)
Total	76 (100)	47 (100)	29 (100)	152 (100)

### Bulk tank somatic cell count

The LnBTSCC geometric mean from all herds in the longitudinal study was 6.13 x 10^3^ cells/mL (Range: 3.61–8.47 x 10^3^ cells/mL). The final mixed model for LnBTSCC included the following variables: frequency of GBS isolation, province, quarter of the year, month as a quadratic term, precipitation measured as mm/month/province, and the interaction between trimester and province. The coefficients of the final mixed model for LnBTSCC are presented in Table 3.

**Table 3:** Final linear mixed model of repeated measures of log-transformed bulk tank somatic cell count for herds in Colombian central western region (n = 152 herds).

Fixed effects variables	Coefficient	P-value	95% Confidence Interval
Intercept	6.09	0.000	5.92; 6.26
Frequency of *S*. *agalactiae* isolation		0.000	
*Never*	Baseline		
*Once*	0.34		0.14; 0.54
*Two or more*	0.62		0.44; 0.81
Province		0.068	
*Caldas*	Baseline		
*Quindío*	-0.41		-0.96; 0.15
*Risaralda*	-0.18		-0.42; -0.04
Quarter of the year		0.000	
*Q1*	Baseline		
*Q2*	0.05		-0.13; 0.23
*Q3*	0.55		0.33; 0.77
*Q4*	0.56		0.32; 0.81
Month	-0.02		-0.07; 0.03
Month^2^^a^	-0.04	0.021	-0.01; 0.00
Precipitation ^b^	0.04	0.003	0.02; 0.07
Interaction quarter of the year and Province		0.000	
Q1*Caldas	Baseline		
Q2*Quindío	0.35		-0.20; 0.89
Q2*Risaralda	0.14		-0.04; 0.33
Q3*Quindío	-0.05		-0.56; 0.46
Q3*Risaralda	-0.24		-0.44; 0.04
Q4*Quindío	-0.10		-0.62; 0.42
Q4*Risaralda	-0.21		-0.36; -0.06
**Random-effects parameters**		**Standard error**	**95% Confidence Interval**
Bulk tank (Variance) ^c^		0.02	0.15; 0.25
Residual: AR4 (ρ4) ^d^		0.04	0.08; 0.22
Residual (Variance)		0.01	0.16; 0.19

^a^ Month as quadratic term.

^b^ Centered and rescaled per 100 mm of rainfall.

^c^ Independent errors and constant variance.

^d^ Fourth level autoregressive structure of errors.

### Cross-sectional study

Even though all selected herds had tested positive for GBS in BTM on 1 to 12 occasions (mean 5.84 ± 3.70) prior to enrollment, GBS was isolated from individual cow milk samples in only 20 of 25 herds. Of 1712 cow-level milk samples, 207 (13%) were GBS positive with a within-herd prevalence from 2% to 59%. In addition, GBS was isolated from rectal swabs in 32 (2%) cows from 8 herds, with a within-herd prevalence from 2% to 20% of the cows. Only two cows were GBS positive both in milk and rectal samples, i.e. half as many as expected based on independence of results from milk samples and rectal swabs. In one herd, GBS was detected in rectal samples, with an intra-herd prevalence of 20%, but not in milk samples. Finally, three (2%) out of 181 environmental samples were GBS positive. The frequency of GBS isolation in the cow-level milk samples, rectal samples, and environment is summarized by herd in Table A in S1 Supporting Information.

### Molecular characterization

Multi Locus Sequence Typing was conducted on 129 BTM isolates from 72 herds (Table 4). Six STs were identified in the BTM isolates (Fig A in S1 Supporting Information), including a new ST (ST1149), which is a single locus variant (SLV) of ST718. The most prevalent STs in isolates from BTM were ST356, ST1, and ST718 (Table 4). The ST distribution differed significantly between the three provinces (P = 0.009) (Table B in S1 Supporting Information). CC61/67 encompassed 67% of the isolates, with ST356 as its most common representative. CC1 and CC103 were represented by ST1 and ST248, respectively (Table 4). Of 45 herds with multiple GBS isolations, less than half (17; 38%) consistently had the same ST, whilst more than half (26; 58%) had two STs and two herds had three STs in BTM (Table A in S1 Supporting Information). Isolates belonged to different CCs in more than half of the herds with multiple STs in BTM (17 of 28).

**Table 4:** Frequency of detection of *Streptococcus agalactiae* clonal complexes (CC) and sequence types (ST)in bulk tank milk (BTM) and cow-level samples from dairy herds in central western Colombia.

Clonal complex	Sequence Type	Longitudinal study		Cross sectional study					
		BTM samples		Composite milk samples		Rectal samples		Environmental samples	
		Isolates	Number	Isolates	Number	Isolates	Number	Isolates	Number
		n (%)	of herds ^a^	n (%)	of herds ^a^	n (%)	of herds ^a^	n (%)	of herds ^a^
CC1	ST1	35 (27)	28	77 (37)	11	5 (16)	1	1 (25)	1
CC61/67	ST61	3 (2)	3	15 (7)	1				
	ST356	37 (29)	18	42 (20)	8	6 (19)	4	3(75)	2
	ST718	31 (24)	21	44 (21)	8	2 (6)	1		
	ST1149	15 (12)	10	4 (2)	3	16 (50)	2		
	ST1175			8 (4)	2				
CC103	ST248	8 (6)	8	12 (6)	3	3 (9)	1		
	ST314			5 (3)	1				
Total isolates		126		207		32		4	

^a^ Number of herds with at least one isolate belonging the respective ST.

In cow-level milk samples, eight STs were detected among 207 isolates. CC61/67 encompassed 54% of the isolates, followed by CC1 with 37%, and CC103 (17%). Five STs were isolated from rectal samples, with ST1149 as the most common type, whilst two STs were detected in environmental samples (Table 4). ST distribution was significant different between sample types (BTM, cow-level milk samples and rectal samples) (P = 0.000).

**Fig 1:**
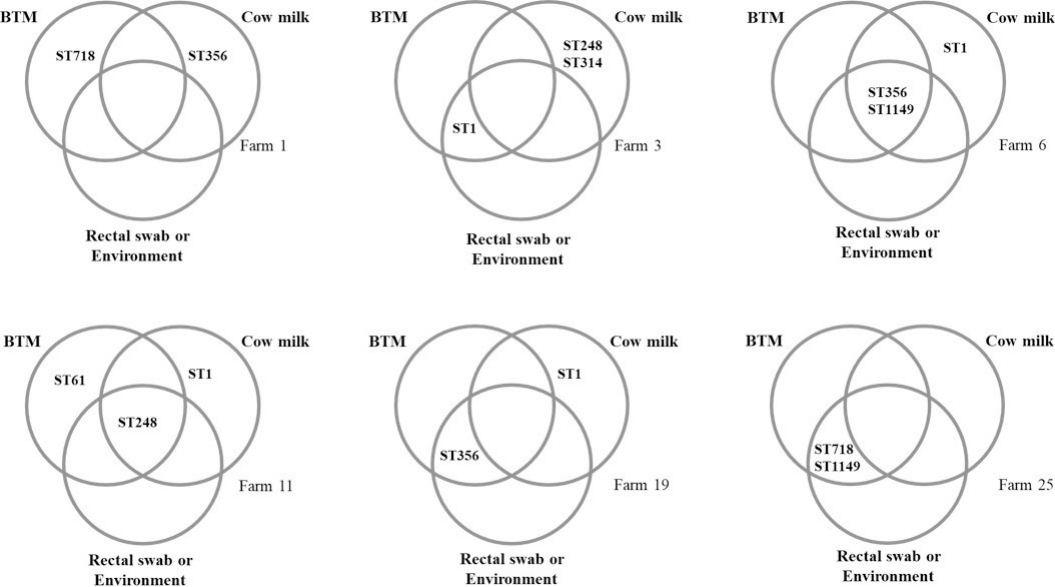
Examples of the distribution of *Streptococcus agalactiae* sequence types (ST) across sample types on Colombian dairy farms. BTM = bulk tank milk. Details of the number of isolates per ST and sample type are given in Table A in S1 Supporting Information.

Strain heterogeneity in cow-level milk samples was higher than in BTM samples, with 2 of 21 herds (9%) yielding as many as 4 STS, 5 (24%) yielding 3 STs, 5 (24%) yielding 2 STs, and only 9 (43%) yielding a single ST. Among the 12 herds with more than one ST, the majority (n = 10) had STs from multiple CCs (Table A in S1 Supporting Information). Being an open herd was significantly associated with the isolation of multiple STs within the same herd (P = 0.018), but not with isolation of multiple CC (P = 0.640). STs that were detected in BTM were not necessarily detected in individual cow milk samples and vice versa (Fig 1). Of 207 GBS isolates from cow-level milk samples, 114 (55%) isolates from six farms belonged to STs that were not isolated from BTM samples. For example, ST356 was detected in 6 cows from Farm 1, but not in BTM, which tested positive for ST718 on one occasion (Fig 1; Table A in S1 Supporting Information). On one farm, GBS was isolated from 16 of 80 (20%) rectal swabs and 3 of 12 BTM samples but not from cow level milk samples and the ST from rectal isolates matched the ST found in BTM (Farm 25, Fig 1; Table A in S1 Supporting Information).

**Fig 2:**
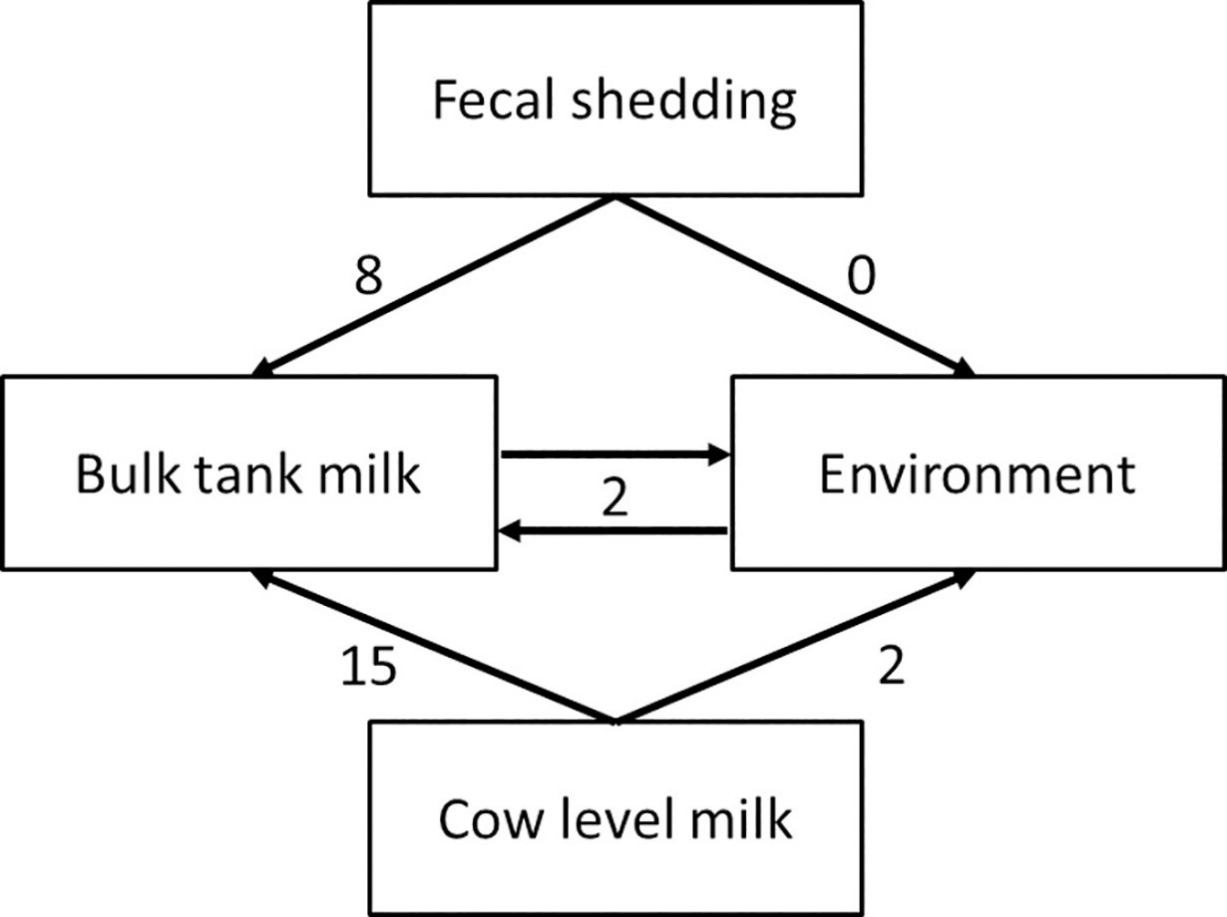
Potential within-farm transmission routes of *group B streptococcus (GBS)*. Numbers over the arrows represent the number of farms where MSLT results support the link.

In another herd, GBS was found in BTM, cow level milk samples and rectal swabs but only the ST from rectal swabs was found in BTM (Herd 19, Fig 1; Table A in S1 Supporting Information). Similarly, in herd 3, the ST isolated from BTM matched the ST from an environmental isolate, but not the ST of isolates from cow-level milk samples. Potential within-farm transmission routes of GBS based on our MLST results are presented in Fig 2.

## Discussion

Results from this study show that GBS epidemiology in the emerging dairy industry in Colombia is different when compared to the traditional dogma of GBS epidemiological characteristics and, characterized by high prevalence, extramammary sources of the bacteria, and high within-farm strain heterogeneity. These results challenge the current understanding of GBS mastitis epidemiology in multiple ways.

### Sporadic isolation of GBS challenges the traditional idea that GBS is a highly contagious pathogen causing chronic infections

Over a period of 6 months, GBS was isolated sporadically in 40% of the positive herds. Given that producers did not receive culture results until the end of the project, and that they rarely use laboratory services to support mastitis control, no specific efforts were made to prevent GBS transmission in the study herds. Contagious transmission would have resulted in high incidence of new infections, which, combined with the presumed chronic nature of *S*. *agalactiae* intramammary infection, would have led to high prevalence of the pathogen and repeated isolation from BTM. Sporadic positivity of BTM samples could result from the introduction of GBS from sources other than milk, such as bovine feces or environmental sources. This has been described for other Gram-positive mastitis pathogens such as *Streptococcus uberis* or *Staphylococcus aureus* [28] and is supported by strain typing data from our study. The detection of the same strain in BTM, rectal swabs and environmental samples suggests that fecal and environmental contamination of teat skin and milking equipment may contribute to presence of GBS in BTM. Transient infection with *S*. *agalactiae*, as described after experimental challenge of cattle with human *S*. *agalactiae* [18], potentially coupled with low shedding could also explain sporadic detection in BTM. Almost 40% of the GBS isolates from cow-level milk samples were ST1, which was the dominant strain shared between humans and cattle [20, 29] and might be related to interspecies transmission. However, without typing data from humans in contact with the cattle sampled in this study is not possible to infer about interspecies transmission or its direction.

### Fecal shedding of GBS is more common than acknowledged

*S*. *agalactiae* was isolated from rectal swabs in a third of the herds in the cross-sectional study, and occasionally in environmental samples, demonstrating that the bacteria can survive in extramammary sources, and that GBS is definitely not an obligate intramammary pathogen. Similarly, in Norway, GBS ST1 and ST130 were isolated from rectal and vaginal swabs of cows and from throat swabs of calves, as well as from environmental samples [17]. Gastro-intestinal carriage of GBS is common in humans, with prevalence of rectal colonization of approximately 30% [30], and carriage is a recognized risk factor for vertical transmission [31]. In 32% of the farms included in the cross-sectional study, the same ST was isolated from BTM and rectal swabs. This finding supports the idea of contamination of BTM by fecal shedding. No evidence of fecal contamination of the environment was found in this study, probably due to the fact that environmental samples collected were from feeders and drinking water only. Sampling of other environmental sources such as floors, alleys and paddocks would support the possibility of environmental contamination and transmission [17]. The role of fecal organisms as cause of mastitis is well-established, particularly for Gram-negative species as *Escherichia coli* or *Klebsiella pneumoniae*, but also for Gram-positive cocci such as *Streptococcus uberis* [32]. We see no reason why transmission via this route would not be possible for GBS.

The novel sequence type ST1149, a single locus variant of the bovine associated strain ST718 was the most prevalent ST among rectal samples, including the herd with high intra-herd prevalence (20%) in rectal samples, but absent in cow-level milk samples. Isolation of new STs was expected because the MLST database contains very limited information from Colombia (https://pubmlst.org). Likewise, there are very few studies on rectal GBS isolates from cattle; therefore, it is difficult to know if the new ST corresponds to the geographic area or the site of isolation. ST1149 could have emerged as a dominant strain in feces from the milk-associated ST718 due to incremental evolution, i.e. small and accumulative genetic changes that lead to adaptability to new environments such as the gastrointestinal tract. This process has been described for GBS ST1, where small genetic changes were associated with significant changes in capsule and pilus production, that allowed it to emerge as an invasive infectious agent in non-pregnant adults [33]. GBS survival or adaptation to extramammary sources may differ between strains, however those differences are difficult to know with the information available until now regarding extramammary sources of GBS. Additional studies in this regard are necessary to know the mechanisms and different level of strain adaptation to extramammary sources.

### Multiple strains per herd are common, which distinguishes emerging from developed dairy industries

High within herd heterogeneity of GBS strains was found in our study as well as recent studies in Colombia [21] and China [34]. This contrasts with results from previous studies where a single strain has been isolated per herd [17, 19, 35, 36]. Most of the studies in GBS epidemiology has been conducted in countries with active GBS control and low GBS prevalence, which is a very different scenario compared to countries with emerging dairy industries. High strain heterogeneity could be related to the biosecurity practices of the herds included. In the USA, purchase of animals to expand the herd has been associated within-herd strain heterogeneity of *Staphylococcus aureus*, another mammary pathogen [37]. In our study area, approximately half of the farmers purchased animals from other herds to expand the herd size, providing a potential explanation for the observed strain heterogeneity. Despite the fact that many herds are open, the ST data showed geographic clustering by province, and a previous study in a different province of Colombia showed a different strain distribution yet again [21]. These results show that GBS molecular epidemiology differs between geographic regions, even in the same country, which could be related to a better adaptation to different environmental and management conditions, or the fact that cattle movements over short distances are more common than large distance, creating strains clusters. In addition to animal movements, poor mastitis control and long-term presence of the pathogen in a herd may lead to gradual development of SLV strain pairs like ST248-ST314 or ST61-ST718 [19].

It is important to highlight individual isolates from BTM samples will not reflect within-herd strain heterogeneity. We characterized up to four BTM isolates per herd and sampled all lactating cows from those herds. Most cow-level milk samples isolates (55%), belonged to strains that were not isolated from BTM samples. For that reason, it was not possible to infer the effects of STs isolated from BTM on BTSCC, as originally intended.

Although analysis at strain level was not possible, our study confirmed that herd SCC is associated with the frequency of detection of GBS. Similar findings have been reported from North America [38] and the Czech Republic [39]. Even when accounting for the presence of GBS, LnBTSCC differed between provinces, which may be related to different commercial demands of the milk processors. In Quindio, the biggest milk buying company uses bonus payments and penalties based on BTSCC, which stimulates dairy producers to control mastitis. By contrast, most herds in Caldas and Risaralda, producers deliver milk to different companies where payments are not based on BTSCC, and this is reflected in higher BTSCC, particularly in Caldas. In addition to GBS status and province, all measures of temporality included in the model were significant (month and quarter of the year). A seasonal effect was also reported by Reyes et al [40], with both their study and ours showing higher BTSCC during the rainy season. Rainfall was similar across departments, and it is not clear how the interaction between quarter, as proxy for rainfall, and department should be explained or interpreted. Seasonal variation in BTSCC has also been reported from other countries [41] but the mechanism underpinning this phenomenon in Colombia needs further study.

In conclusion, high prevalence of GBS was found, both at the herd and cow levels. In this study, GBS was isolated from milk as well as from extramammary sources (e.g. Rectal swabs from cows, and environmental samples) demonstrating that GBS is not always an obligate intramammary pathogen and in some herds, GBS can have a similar molecular epidemiology of an environmental pathogen. Fecal shedding may explain detection of BTM, which was often sporadic. High within-herd heterogeneity of GBS strains was observed in contrast to the situation in fully developed dairy industries. Our findings emphasize that the epidemiology of GBS-causing mastitis is not simply a matter of contagious transmission and chronic infections but differs between production systems, and environmental conditions. The surveillance and control recommendations for GBS should be revised, taking into account its diverse epidemiology and the existence of extramammary sources, to include both contagious and environmental transmission pattern.

## Supporting information

S1 Supporting Information Contains Table A, Table B and Fig A.
(PDF)
