# Supplementary material for: Streptococcus agalactiae is not always an obligate intramammary pathogen: Molecular epidemiology of GBS from milk, feces and environment in Colombian dairy herds
Source: PLoS One. 2018 Dec 10;13(12):e0208990. doi: 10.1371/journal.pone.0208990 (PMC6287850; doi:10.1371/journal.pone.0208990)
Supplement: S1 Supporting Information — Contains Table A, Table B and Fig A. (PDF) [file pone.0208990.s001.pdf]

S1 Supporting Information

Streptococcus agalactiae is not always an obligate intramammary pathogen: Molecular epidemiology of GBS from milk, feces and environment in Colombian dairy herds

Table A. Frequency of group B streptococcus (GBS) isolation and sequence types (STs) isolated in the bulk tank milk (BTM) samples, cow-level milk samples, rectal samples, and environmental samples of the farms included in the cross-sectional study (n=25)

Herd	Lactating cows (n)	Frequency of GBS isolation in BTM [a]	STs isolated from BTM samples	Number of GBS-positive cow-level milk samples	STs isolated cow-level samples	Number of GBS-positive rectal samples	STs isolated rectal samples	Number of GBS-positive environmental samples	STs isolated environmental samples
**1**	165	1	718	6	356	0		0	
**2**	42	11	1	11	1-718-1175	0		0	
**3**	77	7	1	6	248-314	0		1	1
**4**	80	2	718-1149	20	1-718-1149-1175	0		0	
**5**	101	4	1-356	35	1	0		0	
**6**	62	12	356-1149	6	1-356-1149	2	1149	1	356
**7**	72	11	1-356	9	356	0		0	
**8**	75	9	356	19	356	1	356	0	
**9**	26	1	1149	6	1	0		0	
**10**	25	7	1-356	6	1-718	0		0	
**11**	73	8	61-248	13	1-248	3	248	0	
**12**	88	6	718	2	1-718	0		0	
**13**	105	3	1-718	15	718	0		0	
**14**	37	9	61-718	1	718	0		0	
**15** [b]	111	1	61-718	0				0	
**16**	118	4	1	7	1-356	5	1	2	356
**17**	41	11	356-718	8	1-248-356-718	0		0	
**18**	11	5	356	0		0		0	
**19**	45	7	356	2	1	1	356	0	
**20**	17	1	356	0		0		0	
**21**	65	1	356	1	356	2	356	0	
**22**	23	5	356-718	1	356	2	356	0	
**23**	130	6	1-248-1149	33	61-718-1149	0		0	
**24**	43	11	356-718	0		0		0	
**25**	80	3	718-1149	0		16	718-1149	0	
**Total**	1712	146		207		32		3	

[a] number of times that BTM was GBS positive in 12 samplings for six months. [b] Herd where no rectal swabs were collected.

Table B. Sequence type distribution of isolates among the three provinces

Sequence Type	Provinces			
	Caldas	Quindio	Risaralda	Total
1	24	3	8	35
61	3	0	0	3
248	6	0	2	8
356	28	8	1	37
718	21	1	9	31
1149	9	0	6	15
Total	91	12	26	129

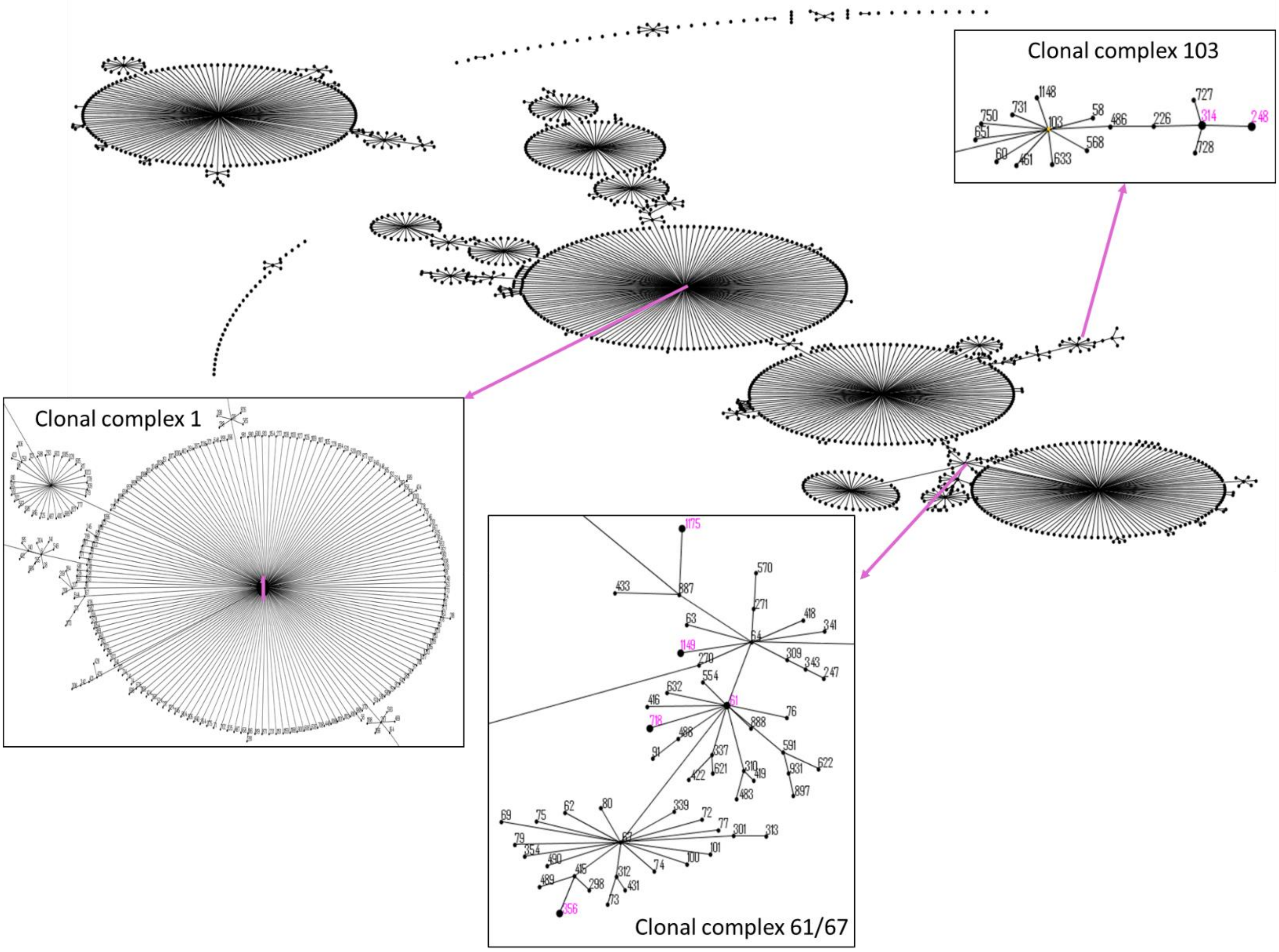

Fig A. The population snapshot for the group B streptococcus isolates obtained from bulk tank milk in this study and the existing in the comparative analysis with the multi-locus sequence typing database. Sequence types in purple were found in this study.
